# Liver transplantation for the treatment of neuroendocrine liver metastases

**DOI:** 10.3389/fsurg.2025.1603704

**Published:** 2025-07-01

**Authors:** Gabriel Orozco, Dharani Ramaiah, Alejandro Cracco, Siddharth Desai, Roberto Gedaly

**Affiliations:** ^1^ Department of Surgery, University of Kentucky, Med Center Health (Bowling Green), Bowling Green, KY, United States; ^2^ Department of Surgery - Transplant Division, College of Medicine, University of Kentucky, Lexington, KY, United States; ^3^ University of Kentucky College of Medicine, Lexington, KY, United States

**Keywords:** neuroendocrine neoplasia, liver metastasases, liver transplant, liver resection, neuroendocrine (D018278)

## Abstract

Neuroendocrine liver metastases (NELM) are commonly observed in patients with advanced neuroendocrine tumors (NETs) and are associated with poor prognosis, primarily due to liver failure and hormone-related complications. While hepatic resection remains the standard surgical approach, orthotopic liver transplantation (OLT) has emerged as a potentially curative treatment in selected patients with unresectable disease. This review summarizes current evidence on the role of OLT in managing NELM, with a focus on patient selection criteria and existing clinical guidelines. Appropriate selection is essential, as improved long-term survival has been consistently demonstrated in patients who meet established eligibility parameters. In conclusion, OLT offers meaningful survival benefits for carefully selected patients with NELM. A multidisciplinary approach and ongoing research into prognostic markers and adjunctive therapies are critical to optimizing outcomes in this challenging clinical setting.

## Introduction

Neuroendocrine tumors (NETs) are diagnosed at stage IV in 66%–85% of patients, and liver metastases are the most common location of metastatic disease (1–4). Neuroendocrine liver metastases (NELM) commonly manifest as multiple lesions affecting both lobes of the liver and are associated with a poor prognosis (5–10). Among patients with NELM, liver failure is the most common cause of death (11–13). Additionally, the production of bioactive amines and polypeptides that circumvent hepatic clearance leads to carcinoid syndrome in 8%–45% of patients, significantly reducing the quality of life (14–16).

Surgical management plays a crucial role in improving survival and reducing symptoms in patients with NELM. Surgical options include anatomic hepatic resections, wedge resections, and orthotopic liver transplantation (OLT) in selected patients. Microwave ablation, transarterial embolization, transarterial chemo and radioembolization, chemotherapy and molecular targeted therapies can be used in patients who are not surgical candidates (17, 18). Peptide Receptor Radionuclide Therapy (PRRT) is a therapeutic approach that uses β-emission radiation to induce tumor necrosis. Y-90 and other radiation therapies have shown promising results; however, they carry risks, including the potential for liver failure. Here, we will review the role of OLT in the management of NELM.

### Liver resection for the treatment of NELM

Liver resection is the cornerstone in the management of NELM as it leads to significant improvement in survival and decreased endocrine symptoms in selected patients. Liver resection improves survival, even if R0 resection cannot be achieved. Studies have shown that a 70% cytoreduction threshold is sufficient to achieve significant symptom control and survival benefits, and this criterion has now been adopted by the North American Neuroendocrine Tumor Society (NANETS) (19–22).

A meta-analysis published by Yuan et al., analyzing data from seven studies and 696 patients, found that the 2-, 3-, and 5-year survival of those undergoing resection were 82%, 74%, and 50%, respectively, compared to 52%, 40%, and 30% in patients undergoing nonsurgical treatments (23). Similarly, a meta-analysis published by Kaçmaz et al., analyzing data from 11 studies and 1,108 patients, reported that surgical resection leads to the best long-term survival compared to no resection, chemotherapy, or embolization (24).

Frilling et al. described three types of NELM based on their radiologic characteristics. Type I refers to a single metastasis, Type II is an isolated metastatic bulk accompanied by smaller deposits, and Type III refers to disseminated metastatic disease. The authors showed that while patients with type I and type II NELM had a 10-year survival of 100% and 75%, respectively, those with type III tumors had a 10-year survival of only 29%. This observation is important for determining which subgroups of patients with NELM may benefit from OLT (25).

### Selection criteria for OLT in patients with NELM

Initially described in 2007, the Milan-NET criteria have proven valuable in identifying patients who may benefit from transplantation. Based on these criteria, the Organ Procurement and Transplantation Network (OPTN) under the United Network for Organ Sharing (UNOS) and the European Society for Medical Oncology (ESMO) have established guidelines to determine transplant eligibility for patients with NELM (Table 1). We will discuss the key factors assessed in patient selection, including tumor histology, resection of the primary tumor and location, absence of extrahepatic disease, extent of liver parenchymal involvement, and age.

**Table 1:** Selection criteria for liver transplant consideration in patients with neuroendocrine tumor liver metastases (28–30).

Characteristics	Milan-NET	OPTN/UNOS	ESMO
Tumor differentiation	Well-differentiated	Well-differentiated	Well-differentiated
Tumor grade and Ki67	Low-grade (G1 or G2)	Low-grade (G1 or G2) Ki67 < 20%	Low-grade (G1 or G2) Ki67 < 10%
Primary tumor location	Primarily gastrointestinal or pancreatic. Tumors with portal system drainage.	Primarily gastrointestinal or pancreatic. Tumors with portal system drainage.	Primarily gastrointestinal or pancreatic
Tumor burden	≤50% liver involvement	≤50% liver involvement	≤50% liver involvement
Prior resection of primary tumor and extrahepatic disease	Yes, at least 6 months prior	Yes, at least 6 months prior to MELD exception request	Yes, at least 6 months prior
Extrahepatic disease	All extrahepatic disease should have undergone resection	No extrahepatic disease at least 3 months prior to MELD exception request. Recheck metastatic workup every 3-months. Patients may come back to the list if any extra-hepatic disease is zeroed and remains stable for at least 6 months	No extrahepatic disease
Extra-hepatic solid organ metastases	All extrahepatic disease should have undergone resection	Permanent exclusion criteria	No extrahepatic disease
Response to prior therapy	Stable disease in response to therapies for ≥6 months	Stable disease in response to therapy for ≥6 months	Stable disease in response to therapies for ≥6 months
Age limit	<60 years	<60 years	<60 years
Imaging requirements	Does not specify	PET scan, SRS, or 68Ga- DOTATATE PET/CT	Does not specify

#### Tumor histology

The tumor's biology is strongly linked to prognosis and must be carefully evaluated when selecting patients for OLT, liver resection, as well as locoregional and systemic therapies. According to the World Health Organization (WHO) NETs can be classified based on their differentiation and proliferation activity. Well-differentiated NETs are G1 (Ki-67 index <2% or less than 2 mitoses per 10HPF), G2 (Ki-67 index 2%–20% or mitotic count 2–20 per 10HPF), or G3 (Ki-67 index >20% or mitotic count >20 per 10HPF). All poorly differentiated tumors are high-grade (G3) with a Ki-67 index >20% or mitotic count >20 per 10HPF (4, 26, 27). The Milan-NET, OPTN/UNOS, and ESMO criteria establish that only patients with G1 or G2 tumor histology should be considered for transplantation (28–30). However, this classification system does not correlate well with liver resection or transplantation outcomes, and the most suitable histologic selection criteria remain a topic of ongoing discussion. In a recent study, Eshmuminov et al. recommended that a 5% cutoff for the Ki-67 index be adopted as a criterion for selecting liver transplantation patients (4). The current recommendation of a 3–6-month observation period after resection of the primary tumor is intertwined with the histologic grading, as it allows the selection of patients with less aggressive tumor biology. In a prior analysis from the OPTN/UNOS database, our group found that longer waiting times were associated with better outcomes and that waiting times greater than 6 months were associated with lower tumor recurrence (25.7% vs. 75.3% (31).

#### Primary tumor resection and location

The Milan-NET, OPTN/UNOS, and ESMO criteria emphasize that surgical resection of the primary tumor must occur before liver transplant consideration. This criterion ensures adequate staging and assessment of the tumor's histologic grade before pursuing OLT. It also guarantees that the primary tumor has been located and that resection with curative intent has been pursued. Mazzaferro et al. also suggested that neuroendocrine tumors (NET) originating from the gastrointestinal tract with portal vein drainage have a higher incidence of liver-only metastatic disease compared to other primaries with hematogenous spread and liver metastases. Accordingly, patients with primary tumors located in the pharynx, esophagus, distal rectum, or extra-gastrointestinal sites are now excluded from OLT by most centers (28–30).

#### Absence of extrahepatic disease

The OPTN/UNOS, Milan-NET, and ESMO criteria recommend that there should not be extrahepatic disease at the time of transplantation. The resection of the primary tumor and all extrahepatic disease should occur at least six months before OLT consideration. Only the OPTN/UNOS guidelines are explicit about extrahepatic solid organ metastases as permanent exclusion criteria for transplantation (Table 1). There is a paucity of data reporting outcomes of OLT in patients who, at their initial presentation, had evidence of extrahepatic disease (32). However, it has been demonstrated that transplanting outside the Milan-NET criteria does not offer significant survival benefits when compared to liver resection (4). In addition, simultaneous resection of the primary tumor at the time of transplantation has been associated with lower overall survival (33).

Common radiologic workup includes multiphasic CT scan, MRI, and different modalities of scintigraphy. Given that 80%–90% of NETs overexpress somatostatin receptors (except for insulinomas), scintigraphy is a key diagnostic tool before transplantation. The OPTN/UNOS selection criteria requires that all patients must undergo PET-scan, somatostatin receptor scintigraphy (SRS), or 68Ga- DOTATATE PET/CT before being considered for transplantation. Etchebehere et al. reported that 68Ga- DOTATATE PET/CT outperforms SRS for detecting well-differentiated NET lesions with a sensitivity of 96% and specificity of 97% compared to 60% and 97% for SRS, respectively. In this series, DOTATE PET/CT was also more sensitive for detecting unknown primary lesions (34). Supporting the OPTN/UNOS recommendation to include some modality of scintigraphy, the study by Albanus et al. found that 68Ga-DOTATATE PET/CT had a sensitivity of 100% for detecting extrahepatic metastases, including bone and lymph node lesions, compared to 47% for CT scan alone (35).

#### Extent of liver parenchymal involvement

The Milan-NET, OPT/UNOS, and ESMO selection criteria exclude patients with liver tumor involvement greater than 50%. As described by Mazzaferro et al., this cutoff is “pragmatical” and has been proven valuable when combined with the other criteria. Supporting data for a cutoff of 50% includes the report by Touzios et al. where patients with NELM affecting more than 50% of the liver had significantly lower 5-year survival (8% vs. 67%, *p* < 0.001) (36). Conversely, the study by Le Treut et al. did not find significant differences in patients transplanted above or below a cut-off of 40% liver involvement. In this study, the authors found that patients with “hepatomegaly” (defined as the enlargement of the explanted liver by 20% or more beyond the patient's normal liver volume determined by Heinemann's formula) had worse 5-year survival (34% vs. 72%, *p* 0.0037) (37). The lack of data comparing outcomes for OLT above and below a cut-off of 50% raises the question of whether there are patients who could benefit from OLT that are being excluded under current criteria.

#### Age

The age range for patients diagnosed with NELM typically spans 47–77 years, with an average diagnosis age of 62 years. Patients undergoing liver transplantation for NELM are, on average, between the ages of 45 and 49 years. In addition to showing improved long-term survival after OLT compared to non-transplant strategies, Mazzaferro et al. analyzed the relationship between patient age and mortality. Patients older than 54 years old were found to have a higher mortality risk associated with comorbidities compared to those who were younger (28). Similarly, our group found that age 45 or younger was associated with significantly better survival compared with those >45 years (70.9% vs. 60% *p* = 0.03) (31). Currently, most guidelines recommend favoring OLT in patients younger than 60 years (Table 1).

### OLT for the treatment of NELM

The role of OLT for the treatment of NELM is still a matter of debate (38). The North American Neuroendocrine Tumor Society (NANETS) guidelines emphasize that, while transplantation yields favorable outcomes in carefully selected patients, its application is limited by the scarcity of donor organs and the requirement for patients to exhibit favorable tumor biology (22).

Several groups have investigated the utility of liver transplantation in patients with liver metastases without extrahepatic disease. Mazzaferro et al. reported a 10-year survival of 88.8% after OLT in patients with NELM fulfilling the Milan-NET criteria compared to 22.4% in the non-transplant group (28). In a subsequent study, they compared OLT vs. liver resection among patients within Milan-NET criteria, finding that OLT was associated with improved 10-year survival (93% vs. 75%, *p* = 0.007) and 10-year disease-free survival (52% vs. 18%, *p* < 0.001) (39). Living donor liver transplantation (LDLT) has been performed for neuroendocrine liver metastases (NELM), though data remain limited. However, emerging evidence suggests that with meticulous patient selection and integration of multimodal therapies, LDLT may offer favorable outcomes in the management of unresectable NELM (40).

Although patients undergoing liver transplants for NELM have shown decreased tumor recurrence compared to those undergoing liver resection, recurrence is still frequent, and it follows a different pattern. Maspero et al. observed that patients undergoing OLT for NELM experienced a higher incidence of multisite recurrences (48%) compared to the resection group (12%), whereas the majority of recurrences in the resection group were intra-hepatic (88%) compared to only 8% in the transplantation group. Sposito et al. studied post-recurrence survival after transplantation and found a median time between transplantation and recurrence of 82.9 months. The most common sites of metastasis were abdominal lymph nodes (59.4%), peritoneum (6.3%), and lungs (6.3%). The only factor associated with decreased post-recurrence 5-year survival in this series was the time from OLT of less than 2 years (0% vs. 89.5%, *p* = 0.001) (41). In an analysis of the OPTN/UNOS dataset from October 1998 to June 2018, our group identified a recurrence rate of 34% among 258 patients transplanted for NELM, with significantly lower recurrence rates observed in those who waited over 6 months for transplantation (31).

## Conclusion

Patients with NELM who undergo OLT demonstrate longer survival rates and extended intervals before tumor recurrence compared to those undergoing resection. The long-term benefits of liver transplantation for NELM are evident primarily in patients who meet the UNOS or Milan criteria. Patients with low-grade NET draining through the portal system, who have had resection of the primary tumor, less than 50% liver involvement, and stable disease for at least 6 months, exhibit a higher likelihood of success. Patients outside these criteria may benefit from other treatment options such as surgical resection, locoregional or systemic therapies. Although numerous studies have found that younger patients experience longer survival rates compared to older patients with similar tumor histology, this age cut-off has varied from 45 to 55 years. Further research should be conducted to study other prognostic factors that benefit or adversely impact long-term outcomes after OLT.

Currently, research has shown that patients who had tumor recurrence after 2 years post-transplant had significantly higher 5-year survival compared to those who experienced early recurrence. Given the complexities in managing these patients, a multidisciplinary approach is recommended to identify appropriate treatment options. The role of liver transplantation for palliation in selected patients with advanced disease should be investigated. Further research is required to determine the most effective combinations of therapies and immunosuppressants that will not only enhance survival rates but also improve patients’ quality of life.
